# Influence of Laser Micro-Texturing and Plasma Treatment on Adhesive Bonding Properties of WC-Co Carbides with Steel

**DOI:** 10.3390/ma17235999

**Published:** 2024-12-07

**Authors:** Tomasz Karol Wojdat, Tomasz Piwowarczyk

**Affiliations:** Department of Metal Forming, Welding and Metrology, Faculty of Mechanical Engineering, Wroclaw University of Science and Technology, 50-370 Wrocław, Poland; tomasz.piwowarczyk@pwr.edu.pl

**Keywords:** sintered carbides, adhesive joints, laser micro-texturing, low-temperature plasma

## Abstract

This article presents research on advanced surface preparation methods for sintered carbides (WC-Co, grade B2) commonly used in the tool industry, particularly in the context of bonding these materials with C45 steel using adhesives. Sintered carbides are widely used due to their high hardness, wear resistance, and good ductility, making them ideal for manufacturing tools operating in harsh conditions. Traditional bonding methods, such as brazing and welding, often result in stresses and cracks. Adhesive bonding has therefore emerged as an effective alternative to mitigate these challenges. The research focuses on comparing the results obtained through modern surface treatment techniques, such as laser micro-texturing and plasma treatment, with traditional methods like grinding, abrasive blasting, and electrolytic etching. The influence of these methods on adhesion properties and the strength of adhesive bonds was evaluated through mechanical tests, including static shear and pull-off tests. An approximately 50% increase in the mechanical strength of adhesive joints was observed for surfaces treated with low-temperature plasma (operating voltage: 18 kV, flow of gasses: 20 l/min., treatment time: 60 s) and laser micro-texturing (infrared fiber laser, wavelength: 1064 nm (±5 nm), power: 20 W), as compared to mechanical grinding. The shear strength of the adhesive joints was equal to 32 MPa and 30 MPa on average in the case of treatment with low-temperature plasma made of helium and argon, respectively. The highest strength of an adhesive joint was equal to 34.5 MPa on average in the case of laser micro-texturing.

## 1. Introduction

Sintered carbides (also called sintered hard metals [1] or hard metals [2]) are currently one of the most dynamically developing industrial materials, owing to their superior hardness and abrasion resistance [3,4], which exceed those of high-speed steels, coupled with good ductility [5], which allows them to work under variable loads at high temperatures in the range of 700–1000 °C [6]. The most common are the tungsten carbide (WC) hard phase, and cobalt (Co) alloy binder phase [7]. Usually, the pressed compacts of tungsten carbide powder with up to 10% of metals such as nickel or cobalt are sintered at about 1500 °C to gain a product with low porosity, very high hardness, and considerable strength [8]. Owing to their unique properties, they are utilized in several industries such as automotive, defense, and aviation, while their main application is in the production of tools, making up about 50% of the raw materials used [9]. Currently, sintered carbides are used primarily for cutting edges of tools used for machining, working tips of mining tools, and plastic working tools [7]. Their use increases the durability of the tools made of them, which significantly affects the efficiency of production processes, and thus allows for satisfactory economic effects [9].

Due to the relatively high cost of sintered carbides, they are typically used in the form of inserts attached to the steel body of tools [10]. There are many techniques for bonding dissimilar materials, ranging from the widely used brazing and high-temperature brazing [7,9] to the resistance welding and fusion welding processes [10,11]. Laser welding of tungsten carbide with steel is also increasingly popular [12,13]. However, the use of the above methods in the process of joining cemented carbides with steel often shows an unfavorable effect of high temperature, which significantly affects the quality of the obtained joints. In the brazing process, the shrinkage of the joint may lead to residual stresses, cracks, and, as a result, the destruction of the joint [9,14]. To prevent this, a shift has been made towards adhesive technology that can minimize the problem of shrinkage stresses [9]. It should be emphasized, however, that it is not possible to change from brazing technology to adhesive technology everywhere, and this is primarily determined by the operating conditions. In the case of the tool working in difficult conditions and transferring heavy loads through them, such a replacement is impossible [9,11].

Adhesive bonding is a modern method of joining, used in the case of joining dissimilar materials that pose several problems due to their different physicochemical properties [9]. Currently, this technology is not only an alternative, but it has developed a stable position and competes with conventional joining methods [15]. In many cases, adhesive bonding provides joints of much higher quality than joints obtained as a result of the use of brazing, welding, or mechanical joining technologies [9]. An adhesive joint, designed and made in an appropriate manner, has several advantages over brazing [9,15,16,17,18]. One of the most important aspects is to minimize stresses and deformations in the joints. This enables the bonding of large surfaces exceeding 1000 mm², while maintaining an even load on the joint. A flexible, thin layer of adhesive takes on unfavorable changes in the linear expansion coefficient of the bonded materials, and seals the joint, protecting it against the harmful effects of moisture and electrochemical corrosion [9]. In addition, the adhesive bonding technology does not use protective atmospheres or vacuum—the entire process usually takes place at room temperature. It is also not necessary to cool the joint or clean it, e.g., from post-flux slag or scale, just like after the brazing process [9,17]. The simplicity of the entire technology is also an important feature—the operator does not have to demonstrate high manual skills, and the entire process can be successfully automated [18]. Examples of the use of the adhesive bonding method in the joints of sintered carbides with steel in the tool industry are, among others, cross-drilling bits, blades in the form of carbide plates glued into the bodies of worm cutters and reamers and turning tools, and carbide scrapers for cleaning conveyor belts [19,20,21].

The possibility of obtaining a durable and functional adhesive joint for sintered carbides, in addition to ensuring the appropriate strength of the adhesive itself, requires the appropriate bonding of the adhesive with the surface of the material being joined (adhesion, wettability) [22,23]. The basic theory of molecular interactions (called the physical–chemical theory) describes and explains the adhesion phenomenon in the most comprehensive way, recognizing it as the result of the interaction of intermolecular forces (van der Walls, dispersion, dipole, or induction), occurring between the molecules of the adhesive and the joined materials. In the case of mechanical adhesion, the strength of the joint is determined by the strength of the adhesive, which penetrates unevenness of the surface, anchoring itself firmly in the recesses of the material with a rough surface and is absorbed by the porous substrate during application. The degree of penetration depends mainly on the viscosity of the adhesive, temperature, pressure, penetration time, and cross-section and depth of the recesses. Cobalt provides the brittle WC with excellent wetting and adhesive properties [19,24]. Regardless of the selected bonding technology, it should be remembered that sintered carbides are materials that are difficult for wetting, both by braze filler metals and adhesives, so they should not be joined without removing the surface layer after sintering. Hence, the appropriate choice of method of preparing sintered carbides’ surface for adhesive bonding is so important, because it determines the functionality of adhesive joints.

The surface of sintered WC-Co carbide plates is treated with low-temperature plasma and laser micro-texturing before the adhesive bonding process. Plasma treatment is a well-known method frequently used to activate polymer materials, improving specific adhesion and increasing the strength of adhesive joints as a result. Rymuszka et al. [25] studied the modification effect in a PEEK (polyether ether ketone) surface of air plasma treatment. It was observed that the changes were due to the emergence of polar groups over the PEEK surface and the surface roughness, contact angle, and surface energies. For example, the lowest contact angle observed was equal to 11.7° as compared to 83.4° for unmodified PEEK surfaces [25]. Sundriyal et al. [26] showed a large impact of plasma treatment produced from various gasses (air, oxygen, ammonia, argon) on the strength of PEEK adhesive joints. For example, the shear strength of argon plasma adhesive joints was equal to 33 MPa compared to 17 MPa for untreated surfaces [26]. The effect of modifying the surface of sintered carbides using low-temperature plasma, especially for the adhesive bonding process, is not yet well known and described in the literature.

Nowadays, laser micro-texturing is a more and more common method of treatment of various materials such as polymers, metals, and composites, for various applications. There are also known applications of laser micro-texturing of sintered carbides, but they are mainly aimed at increasing the durability of carbide blades in cutting tools. For example, during machining processes, a high temperature is generated in the cutting zone due to plastic deformation, which causes reducing the lifetime of cutting tools. Guimaraes et al. [27] were investigating that laser micro-texturing causes an increase in tool wettability (reduction wetting angle of 27%), providing an enhanced lubrication effect, a reduced tool–chip friction, and a lower tool wear rate [27]. Yilbas et al. conducted research on laser micro-texturing to increase the hydrophobicity of SiC carbides. They showed that the laser-treated surfaces consist of fine grooves and pillars and that the resulting surface roughness enhances the surface hydrophobicity [28]. Furthermore, the fracture toughness of the treated surface is reduced possibly because of the microhardness increase at the surface [28]. Also, Fang et al. in paper [29] describe the use of laser micro-texturing as an effective method for improving the tribological properties of sintered carbides in terms of increasing the lifetime of cutting tools.

There is limited information in the literature regarding the effect of laser micro-texturing on mechanical adhesion in sintered carbide adhesive joints. There is a known solution of using laser heating to increase the adhesion forces between hard coatings and sintered carbides. The results indicate that a significant increase in adhesion force can be achieved for the laser beam power density of up to 4100 W/cm^2^, for monolayer (single—Ti(C,N) and double—Ti(C,N)+TiN) coatings only [30]. Additionally, laser texturing is used in biomedicine applications. In paper [31], the main goal of laser surface micro-texturing techniques in biomedicine was indicated, which is to increase cellular activity on the implant surface. In bone remodeling, textured surfaces provide a greater surface area for integrating the implant into the bone through the osseointegration process. Furthermore, textured surfaces also enable tissue ingrowth and promote the mechanical stability of implants [32]. Work [33] describes the use of laser micro-texturing to prepare steel surfaces intended for adhesive bonding. The influence of laser micro-texturing on the properties of adhesive joints made of 30CrMnSiA steel was examined. The authors made three patterns (dimple, groove, and grid) on a 30CrMnSiA steel substrate using a nanosecond fiber laser. The shear strength of adhesive joints in the green state averaged 7.3 MPa and was significantly improved for joints after laser micro-texturing. For the groove-type dimple pattern, it was equal to 14.6 MPa and for the mesh-type pattern, it was equal to 23.3 MPa. For the grooved and meshed samples, there was an increase of 219% and 348%, respectively, compared to the as-prepared samples. The failure mechanism of the joint also changed from adhesive in the non-treated state, to mixed for the groove pattern, and to cohesive for the mesh pattern [33].

Despite extensive prior research on adhesive bonding of WC-Co carbides, limited studies have focused on optimizing surface preparation methods for Agomet F330 to further enhance its bonding performance. For instance, Piwowarczyk et al. [34] demonstrated that Agomet F330 provided higher bonding strength compared to epoxy- and polyurethane-based adhesives, primarily due to its excellent wetting characteristics and strong adhesion to metallic surfaces. Furthermore, Mirski et al. [35] confirmed its superior durability under dynamic loads, making it a preferred choice for applications involving high mechanical stress. These findings supported the selection of Agomet F330 as the adhesive for this research, as it consistently outperformed alternatives in terms of strength and reliability. This study aims to bridge this gap by evaluating modern surface treatment techniques, such as low-temperature plasma processing and laser micro-texturing, to maximize the adhesion and mechanical strength of joints. By addressing this knowledge gap, the research contributes to the development of more efficient and reliable adhesive technologies for industrial applications. The results were compared with traditional WC-Co carbide surface preparation methods such as grinding, abrasive blasting, and electrolytic etching [9,34,35,36,37].

## 2. Materials and Methods

**Figure 1:**
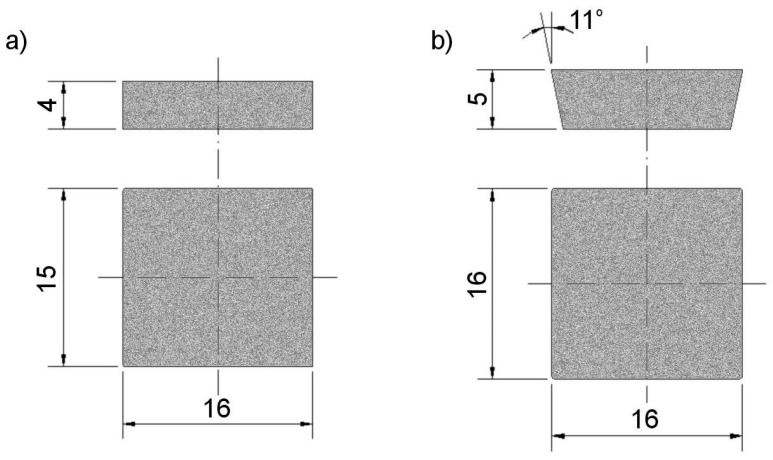
Shape and dimensions of WC-Co carbide grade B2 plates for static tests: shear (**a**) and pull-off (**b**).

The cutting inserts for tools are made of various grades of sintered carbide. Due to the grain size, chemical composition, and properties, they can be divided into individual groups, each of which has a separate application. For the tests, WC-Co carbide plates, grade B2 (group B—used for mining tools), were used, with a content of 91 wt% WC (tungsten carbide) and 9 wt% Co (cobalt matrix). The selected plates are characterized by coarse WC tungsten carbide grain (1–7 µm) evenly distributed in the cobalt matrix, having a hardness of 1220 HV and a bending strength of 2300 MPa [38,39,40]. We used two different shape types of plates, selected for carrying out static shear (Figure 1a) and pull-off (Figure 1b) tests. Two types of plate shapes were selected due to the different methods of grip attachment for the shear and pull-off tests. The adhesive bonding surface area of the carbides for the shear and pull-off tests was 240 mm² and 210 mm², respectively.

WC-Co carbide plates were bonded to metal bodies with a cross-section of 16 × 16 mm and a length of 80 mm. Medium-carbon steel for quenching and tempering, grade C45, was selected for the bodies. The C45 steel is characterized by high strength (Rm = 560–850 MPa) and high ductility (A_5_ = 14–17%) [41]. It is used in joints with sintered carbides, because thanks to its high yield point, the possibility of cracks in carbide fittings is eliminated. The adhesive used for bonding WC-Co carbides to C45 steel was Agomet F330, a methacrylic resin with high shear strength and excellent durability. Previous research by Mirski et al. [34,35] highlighted the adhesive’s compatibility with WC-Co carbides, while Piwowarczyk et al. [36] compared its performance with other adhesive types, concluding that Agomet F330 offered the highest mechanical strength and stability. Additionally, it showed better resistance to environmental factors such as humidity and temperature variations [37]. Based on these studies, Agomet F330 was identified as the most suitable candidate for this research. According to the manufacturer’s data, the adhesive enables joints with a shear strength of up to 40 MPa to be obtained. During bonding, a constant value of the gap of 0.2 mm was established by means of spacer wires. The authors studied, i.e., the influence of the surface preparation of sintered carbides by mechanical and chemical methods, and the obtained results constitute a good point of reference for the discussion of the obtained results.

**Figure 2:**
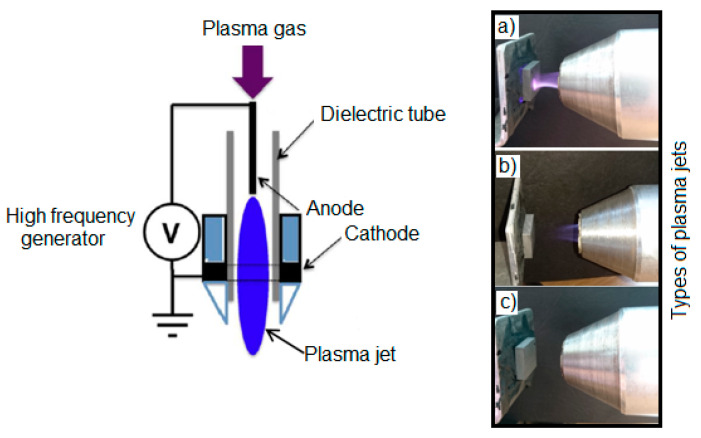
Schema of a device for the low-temperature plasma treatment and types of plasma jets depending on plasma gas: He (**a**), Ar (**b**), and N_2_ (**c**).

The surface of the WC-Co carbide plates was treated with low-temperature plasma produced from various gasses: Ar, He, N_2_, and Ar + CO_2_ (82% Ar and 18% CO_2_). The treatment was carried out using a 300 W device, the operating voltage was 18 kV, and the flow of individual plasma gasses was 20 l/min (University of Science and Technology, Mechanical Department, Wroclaw, Poland). The treatment time was 60 s in each case. The operating principle of the plasma-generating device is shown (Figure 2). During the treatment, depending on the type of gas used, significant differences in the appearance of the plasma jet formed could be observed. The most pronounced jet was from helium (Figure 2a) followed by argon (Figure 2b). In the case of nitrogen (Figure 2c) and the Ar + CO_2_ mixture, the plasma jet was almost invisible.

Laser processing was carried out using an MOPA 4 infrared fiber laser equipped with a galvoscanner with an f-theta lens (DK Lasertechnik, Krakow, Poland) with a useful repetition frequency range of 20 to 600 kHz. This is a pulsed laser with a wavelength of 1064 nm (±5 nm) and a power of 20 W. The organization of work at the research stand consisted of implementing various surface treatment trajectories (parallel lines, grid, etc.), and then performing processing for various process parameters of the laser–power, speed, and number of transitions. The influence of various process variables was assessed by measuring the surface roughness profiles of the machined sintered carbide samples. Surface roughness was measured using a MarSurf PS10 contact profilometer (Mahr, Göttingen, Germany) according to the ISO 21920-3 [42] guidelines. The device is equipped with an inductive skid-measuring head with a tip radius of 2 µm and a measuring force of approximately 0.7 mN. Additionally, a 3D map of the obtained profiles was made using an Olympus laser microscope, model 3D LEXT (Olympus, Tokyo, Japan). The fractures of adhesive joints after the shear test were examined using scanning electron microscopy (SEM). The studies were performed using a Tescan Vega 3 scanning electron microscope (TESCAN ORSAY HOLDING, Brno-Kohoutovice, Czech Republic).

Immediately after low-temperature plasma and laser micro-texturing treatment, the surface free energy (SFE) and its polar and dispersion components were determined for each sample. Measurements were performed using a Krűss DSA-HT12 goniometer (Kruss GmbH, Hamburg, Germany), equipped with a video camera, and a computer system (DSA3) for visualizing measurements and recording drop dimensions. The SFE was determined using the Owens–Wendt (OW) method, based on the measurements of the wettability angles, using two measuring liquids. According to a literature review [42,43,44,45], the measurements were made using the two most commonly used liquids—distilled water (polar liquid) and diiodomethane (nonpolar liquid). The properties of selected measuring liquids are presented in Table 1.

**Figure 3:**
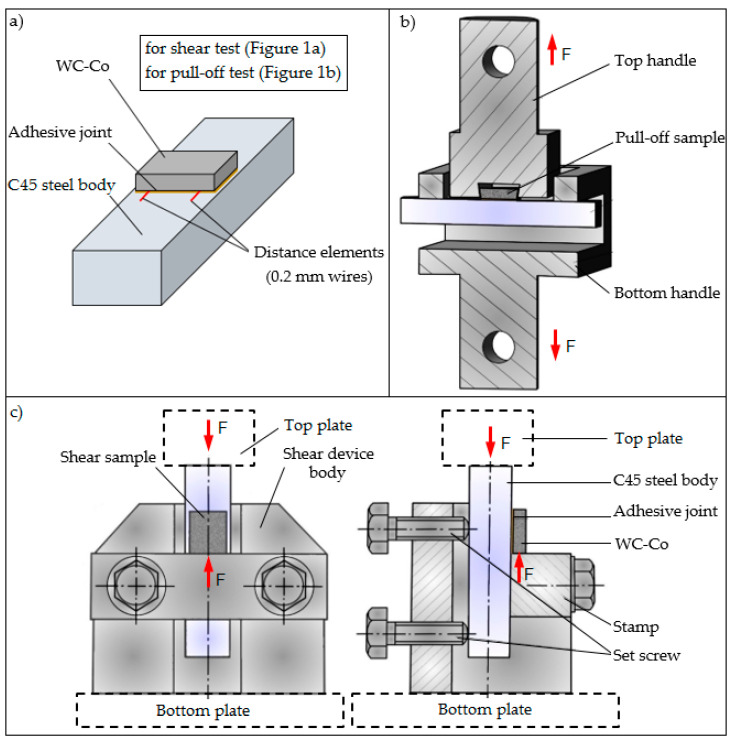
Model of WC-Co–C45 steel adhesive joints (**a**); instrumentation and method of fixing samples of adhesive joints for pull-off (**b**) and shear (**c**) tests.

Mechanical tests were carried out on an Instron 3369 (Instron, Norwood, MA, USA) testing machine, with a maximum force of 50 kN, and the obtained results were recorded using Bluehill 2 software. The speed of the crossbeam of the testing machine was 2 mm/s in both cases. Due to the variable geometry of carbide fittings, the strength tests of their connections are not standardized; therefore, the tests were carried out only based on selected guidelines of the EN 1465:2009 [46], EN 2243-1:2006 [47], and EN 2243-2:2006 [48] standards. Compressive shear and tensile pull-off tests were performed on the adhesive joints (Figure 3a). The instrumentation and method of fixing the samples of adhesive joints for the pull-off and shear tests are shown in Figure 3b and Figure 3c, respectively.

The samples for the tensile and shear tests differed only in the shape of the WC-Co plate (Figure 1a,b). The WC-Co plate for the tensile test had its lateral surfaces beveled at an 11° angle to allow for mounting in a specialized fixture (Figure 3b) and to generate tensile stresses in the adhesive joint. The fixture (along with the sample) was then secured in the grips of an Instron 3369 testing machine, where it was subjected to tensile loading (pull-off test under tension).

In the case of samples for the shear test, the WC-Co plates had lateral surfaces perpendicular to the adhesive surface (Figure 1a). The sample was also mounted in a specialized fixture (Figure 3c) in such a way that the WC-Co plate rested against the fixture’s stamp, while the steel body extended above the height of the fixture. The fixture, along with the sample, was then placed on the bottom plate of the testing machine, and the top plate was pressed against the steel body’s surface. This generated shear stresses in the adhesive joint (shear test under compression). Each test was stopped upon the failure of the adhesive joint.

## 3. Results and Discussion

### 3.1. Surface Properties of Sintered Carbides After Low-Temperature Plasma Treatment

The task of low-temperature plasma treatment was primarily to modify the surface properties of WC-Co carbides, such as SFE and its components (polar and dispersive). These are important parameters from the point of view of obtaining strong adhesive bonds in adhesive joints (specific adhesion). In addition, this treatment has a negligible effect on changing the surface structure, and thus on mechanical adhesion. The roughness parameters (Ra, Rz) before and after processing were very similar and within the measurement error range. The average value of the Ra parameter was equal to 1.25 ± 0.15 µm, and the Rz parameter was equal to 8.52 ± 0.71 µm.

**Figure 4:**
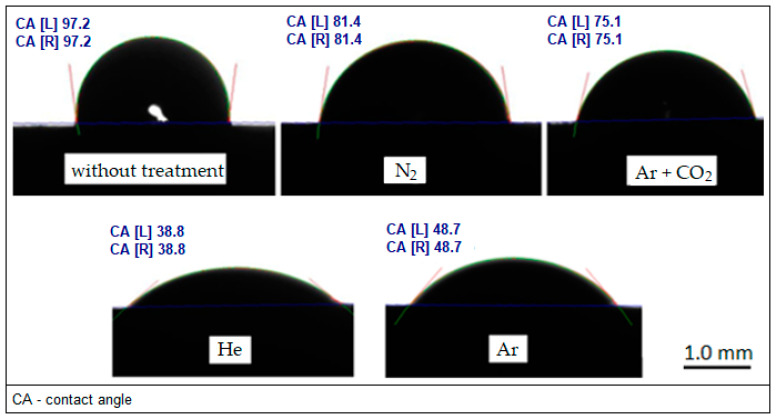
Examples of wettability of WC-Co carbide surfaces with distilled water before and after low-temperature plasma treatment generated from various gasses.

Firstly, which of the tested plasma gasses (Ar, He, N_2_, Ar + CO_2_) has the highest influence on the change in the surface properties of WC-Co carbides was checked. Using a goniometer, the values of wettability angles were determined. The method of measuring contact angles on sample WC-Co carbides before and after plasma treatment with distilled water is shown in Figure 4.

**Figure 5:**
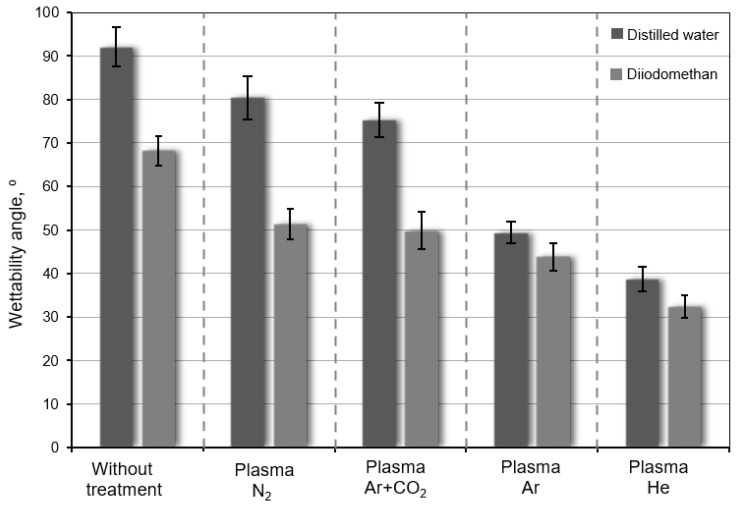
The wettability angle average value of WC-Co carbides before and after low-temperature plasma treatment.

The wettability measurement results are presented in the form of a bar chart with standard deviation (Figure 5). These are the average values from five measurements for each of the gasses from which the low-temperature plasma was generated. As the surface free energy (SFE) of the measuring liquid decreased, lower values of wettability angles were obtained.

The application of low-temperature plasma treatment produced from inert gasses, such as helium and argon, has a significant impact on improving the wettability of the sintered carbide surfaces with the measuring liquids selected for testing. In the case of other gasses such as N_2_ and a mixture of Ar and CO_2_, this effect is much smaller, which may indicate insufficient effectiveness of the introduced modifications.

Secondly, based on the measurements of the wettability angles (Figure 4) of the WC-Co carbide grade B2 surface with the measuring liquids presented in Table 1, the values of the SFE (Table 2) and its components were determined. These values were determined automatically by the DSA3 firmware according to the Owens–Wendt (OW) calculation algorithm. In the OW method, the surface free energy ($\gamma_{S}$) of the solid is the sum of the polar ($\gamma_{S}^{p}$) and dispersive ($\gamma_{S}^{d}$) components (1) [42,43,44,45].

(1) $$\gamma_{S}=\gamma_{S}^{d}+\gamma_{S}^{p}$$

In the case of measurements of the contact angle θ tangential to the outline of the droplet at the point of contact of three phases, solid, liquid, and gas, by a measuring liquid with a known SFE value ($\gamma_{L}$) and polar ($\gamma_{L}^{p}$) and dispersion ($\gamma_{L}^{d}$) components, Equation (2) is used.

(2) $$\gamma_{L}(1+cos\theta )/2={\left(\gamma_{S}^{d}+\gamma_{L}^{d}\right)}^{0.5\;}-2{\left(\gamma_{S}^{p}+\gamma_{L}^{p}\right)}^{0.5}$$

Since there are two unknowns in Equation (2), $\gamma_{L}^{p}$ and $\gamma_{L}^{d}$, it is insufficient to determine the desired SFE value. For this reason, wetting angle measurements should be performed using two different measuring liquids, which allows for the formulation of an equation of the same form as (2) but with different values of constant coefficients.

Similarly, the use of helium as a plasma gas has the greatest impact on the increase in SFE by over 60% and in the polar component by 480%. A significant improvement in these values can also be seen when using argon as plasma gas by more than 40% and 330%, respectively. In the case of the other gasses used in the tests (N_2_ and mixture of Ar + CO_2_), the influence of plasma treatment on the surface properties of WC-Co carbides was poor. The significant increase in the SFE and its components indicates that the modification of the surface using low-temperature plasma generated from inert gasses such as Ar and He is effective and should be expected to have a beneficial effect on the mechanical properties of the adhesive joints of sintered carbides with steel.

### 3.2. Surface Properties of Sintered Carbides After Laser Micro-Texturing

The task of laser processing was to obtain a well-developed real surface of WC-Co carbides, which increases the contact surface of the adhesive particles with the substrate and is the cause of greater intermolecular interactions, and thus an increase in adhesive properties [9,23]. It was assumed that the roughness should be in the range of Ra = 3–4 µm and Rz = 10–15 µm [34,46]. Greater roughness promotes the formation of notches and limited wetting by adhesives with increased viscosity, causing air to be trapped in the cavities [9,38]. As roughness increases, the surface may exhibit hydrophobic to superhydrophobic properties [9]. Lower roughness may, in turn, be insufficient to obtain high mechanical adhesion.

First, a window of laser-operating parameters enabling effective processing of sintered carbides was determined. For a constant machining speed of 400 mm/s, a power window in the range of 20–30% (4–6 W) and a frequency of 100 Hz were determined. First, laser processing was performed for three patterns—parallel lines and a grid. In each case, the spacing between the lines was equal to 0.1 mm. Ultimately, a grid pattern was chosen to make the adhesive joints. In paper [46], it was shown that this type of texture can achieve the best surface adhesion properties (best wettability and mechanical strength of adhesive joints). Additionally, the texture in the form of parallel lines has anisotropic features, so high shear strength can be obtained when the force acts in a direction perpendicular to the cut lines. It was empirically determined that the assumed roughness could be obtained after 10 repetitions of the laser operation, for a power of 25% (5 W).

**Figure 6:**
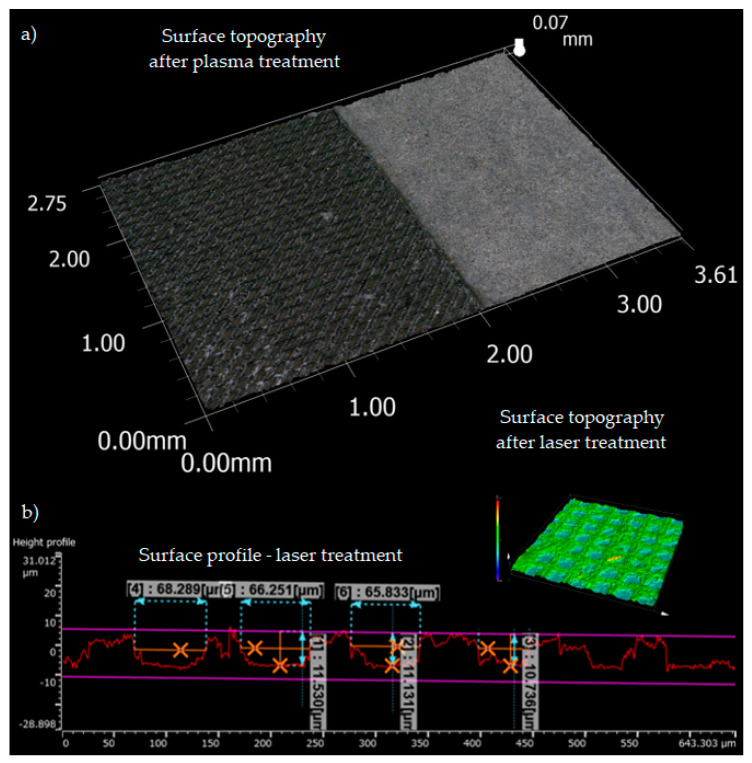
Surface profile of WC-Co carbide after laser and plasma treatments (**a**); example of measuring width (w) and depth (h) of grid texture (**b**).

The grid texture allows for obtaining a well-developed surface with numerous cavities, creating good conditions for obtaining strong mechanical adhesion. Low-temperature plasma treatment does not change the surface topography of WC-Co (Figure 6a). The effects of processing were assessed by measuring surface roughness using contact methods (profilometer) and non-contact methods (laser microscope). The parameters (width (w) and depth (h)) of the cut groove were also measured on a microscope (Figure 6b).

For the laser-operating parameters of power: 25% (5 W), frequency: 100 Hz, speed: 400 mm/s, and number of repetitions: 10, the average value of the roughness parameters Ra and Rz was equal to 3.25 ± 0.22 μm and 12.60 ± 1.85 μm, respectively. The groove width and depth were in the range of 65–67 μm and 10–12 μm, respectively.

The comparison of the surface roughness parameters (Ra, Rz) of WC-Co carbides obtained for different treatment methods is shown in Table 3—these are average values from five measurements.

Surface properties, such as wettability and surface free energy (SFE), of the WC-Co carbides are significantly improved after laser micro-texturing. The surface wettability is enhanced, with average contact angle values for diiodomethane and distilled water measured at 34.6° ± 3.8° and 41.2° ± 5.1°, respectively. The SFE after laser treatment increased significantly, reaching 63.3 mJ/m², with dispersive and polar components of 42.8 mJ/m² and 20.5 mJ/m², respectively. These values are comparable to those obtained through low-temperature plasma treatment using inert gasses such as Ar and He.

The enhanced surface of WC-Co carbides after laser micro-texturing provides favorable conditions for achieving strong mechanical adhesion. The resulting surface irregularities (grid grooves) serve as effective interlocking for adhesive particles. Plasma treatment has a negligible impact on altering the surface structure of WC-Co carbides. However, it significantly increases the surface free energy (SFE) and its components (polar and dispersive). As shown in Table 2, the SFE of WC-Co carbides increases from 41.7 mJ/m² before plasma treatment to 58.8 mJ/m² and 66.9 mJ/m² after Ar and He plasma treatments, respectively. The dispersion component rises from 36.8 mJ/m² before plasma treatment to 42.5 mJ/m² and 43.1 mJ/m² after Ar and He plasma treatments, respectively. The most significant increase is observed in the polar component, which grows from 4.9 mJ/m² before treatment to 16.3 mJ/m² and 23.8 mJ/m² after Ar and He plasma treatments, respectively. The increased chemical activity of the surface following plasma treatment is advantageous for enhancing specific adhesion, thereby improving the mechanical strength of adhesive joints.

### 3.3. Mechanical Properties of Adhesive Joints

The evaluation of the surface modification of WC-Co carbides using low-temperature plasma generated from He and Ar was also conducted based on the study of the mechanical properties of adhesive joints. Sets of adhesive bonds were made for each surface treatment method—low-temperature plasma generated from He and Ar, mechanical grinding, electrolytic etching, and laser micro-texturing. The parameters of mechanical grinding and electrolytic etching were selected based on the literature [34]. Mechanical grinding was carried out on a diamond wheel with a grain equal to 30 μm. In the electrolytic etching process, a 4-molar sodium hydroxide solution (NaOH) was used as the electrolyte. The electrode voltage was equal to 3500 mV and the etching time was equal to 10 min.

**Figure 7:**
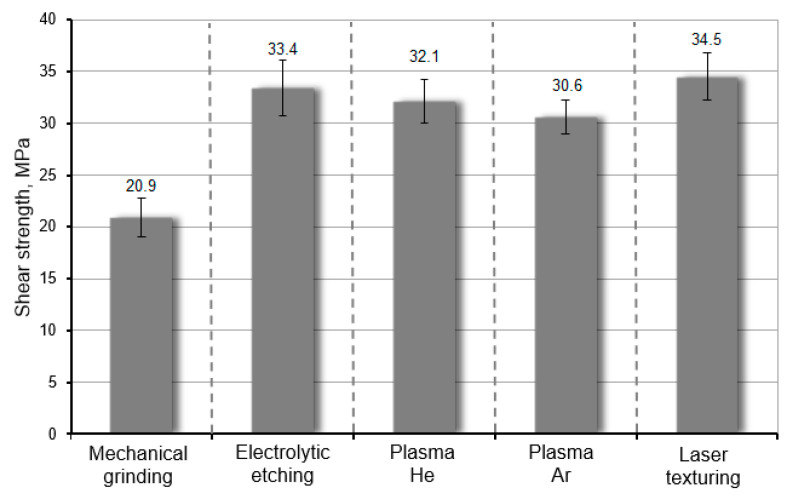
The results of shear strength of WC-Co–C45 steel adhesive joints depending on the method of surface preparation.

Two types of WC-Co–C45 steel adhesive bonds were made—for the static shear test and for the static pull-off test. The static shear strength test of adhesive joints was carried out in accordance with the guidelines of EN ISO 13445 [47], on the geometry of samples and measuring instruments. During the tests, the samples were fixed in the instruments shown in the previous section (Figure 3). The results from the static shear test for the various surface preparation methods are presented in Figure 7. The results for the surface preparation methods, mechanical grinding and electrolytic etching, come from the authors’ earlier publication [34]. These are average values of five measurements, and the error bars represent the standard deviation. The surface of adhesive joints was the same like WC-Co carbide surface and was equal to 240 mm^2^.

**Figure 8:**
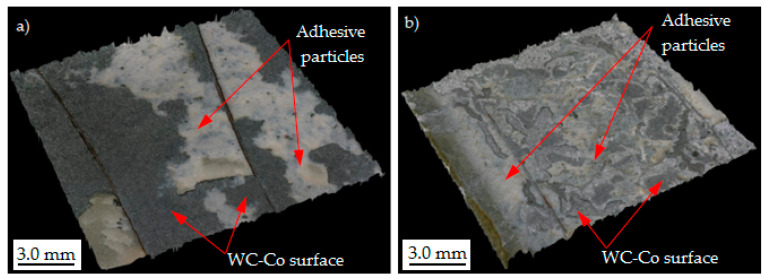
Mixed adhesive–cohesive (AF+CF) fracture mechanism of WC-Co -C45 steel adhesive joints after shear test. Laser (**a**) and plasma (**b**) treated surfaces of WC-Co carbides.

Analyzing the results obtained based on the static shear test, the achievement of high mechanical strength of WC-Co–C45 steel adhesive joints is primarily determined by obtaining a sufficiently high specific adhesion, which is determined by obtaining a large surface free energy (SFE). The application of mechanical treatment, which is mainly used to obtain an appropriate surface expansion and thus increase the mechanical adhesion, is not sufficient in the case of WC-Co carbides. This is due to their high hardness, which makes it difficult to obtain a sufficiently large surface expansion and does not bring the desired effects. Chemical treatments, such as electrolytic etching or low-temperature plasma treatment, yield much better results. The slightly higher shear strength in the case of electrolytic etching compared to the low-temperature plasma treatment is because, in addition to good surface cleaning, it also allows for its significant expansion [46]. As described in [34], the application of this treatment increases the surface roughness parameters, such as *Ra* from 1.8 to 2.3 µm. Similar roughness was obtained using laser micro-texturing, the additional advantage of which is the ability to precisely control the stereometry of the processed surface. The use of laser processing allows for obtaining adhesive joints with high shear strength, which had an average value of 34.5 MPa. The fracture mechanism of adhesive joints was a mixed adhesive–cohesive type (AF+CF) (Figure 8a). In the case of treatment using low-temperature plasma generated from Ar and He, the fracture mechanism of the adhesive joints was also mixed (AF+CF) (Figure 8b).

**Figure 9:**
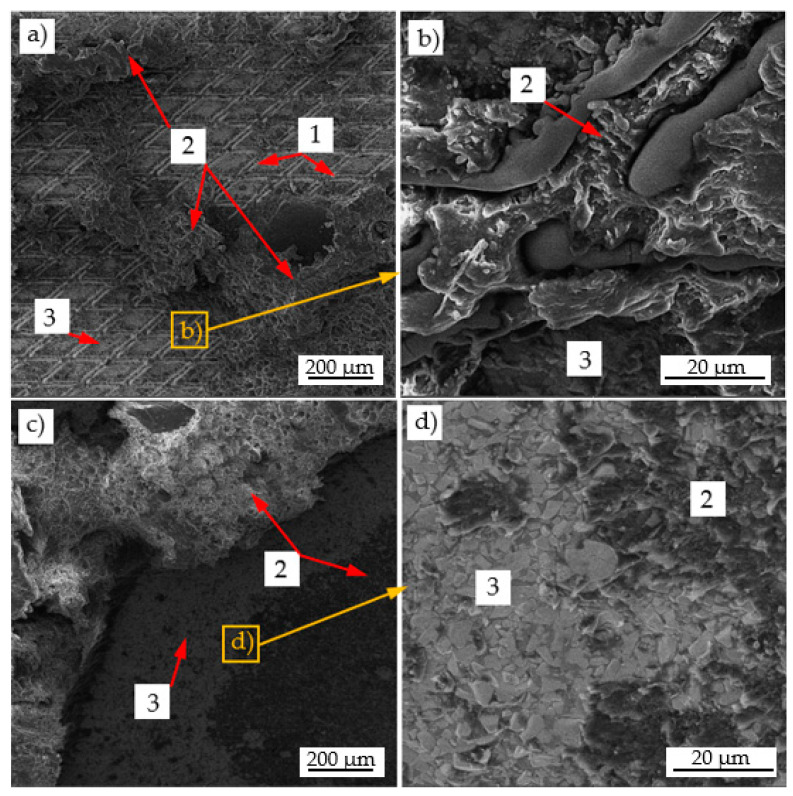
SE (Secondary Electron) image of adhesive joint failure after shear test. WC-Co carbide after laser treatment (**a**,**b**) and WC-Co carbide after plasma treatment (**c**,**d**): 1—laser grooves, 2—adhesive particles, and 3—WC-Co carbide surface.

The grooves (1) formed during the laser processing created favorable conditions for effective mechanical interlocking of adhesive particles (2) (Figure 9a). The adhesive particles (2) filled the cut grooves and anchored well within them (Figure 9b). During the shear test, a cohesive fracture (CF) of the adhesive was observed at the groove locations, while at the non-laser-treated locations, the adhesive particles detached from the WC-Co carbide surface, indicating an adhesive fracture mechanism (AF).

In contrast, the use of low-temperature plasma did not alter the surface roughness parameters. The observed increase in the mechanical strength of adhesive joints was attributed solely to enhanced specific adhesion forces. Joint failure occurred due to a combination of partial adhesive decohesion (CF) and adhesive separation from the WC-Co carbide surface (AF) (Figure 9c,d).

In summary, plasma treatment generates a smoother interface and increases the surface free energy (SFE) primarily by introducing polar functional groups; laser micro-texturing significantly improves mechanical interlocking by creating grooves and surface irregularities that allow the adhesive to anchor more effectively. This mechanical interlocking complements the chemical adhesion provided by the adhesive, resulting in superior overall adhesion strength. Furthermore, laser micro-texturing increases the effective surface roughness (Table 3), which promotes better spreading (wettability) of the adhesive, particularly for high-viscosity adhesives like Agomet F330. The grooves act as channels for adhesive flow, enhancing penetration and reducing the likelihood of air entrapment at the interface. While the plasma-treated surface offers a larger areal spread, its relatively smooth nature does not provide the same level of mechanical anchoring, which is critical for achieving high shear strength in adhesive joints (Figure 7).

Obtaining greater strength of the adhesive joints depends on the introduction of additional modifications of the adhesive joint. As shown in [35], it is possible to obtain adhesive joints with a shear strength of up to 55 MPa, obtained because of increasing the cohesive forces in the adhesive joint in combination with an appropriate method of preparing the surface of WC-Co carbides. This is possible due to the composite structure of adhesives, i.e., an adhesive base enriched with filler—an additional phase of a different size, type, and content [9,35].

**Figure 10:**
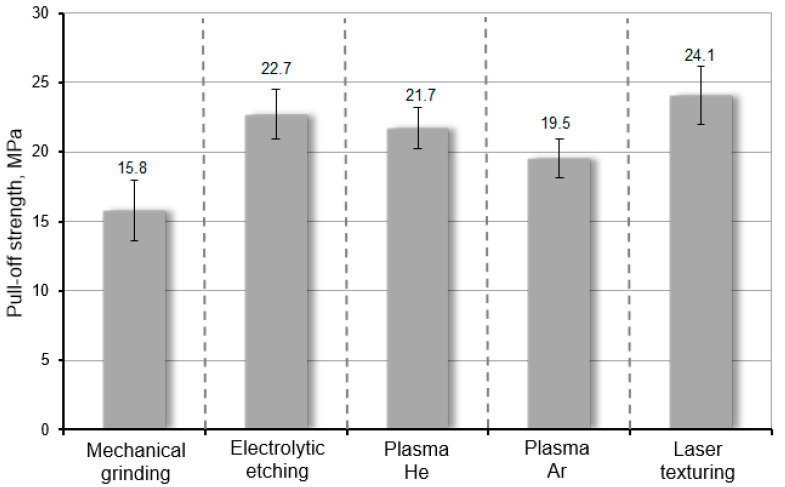
The pull-off strength of WC-Co–C45 steel adhesive joints depending on the method of surface preparation.

The pull-off strength measurements were carried out in accordance with the requirements of the EN 15870 standard [48]. The samples were made in the same way as in the case of the samples for the static shear test. The results from the static pull-off test of the adhesive joints depending on the method of the preparation of the WC-Co surface are shown in Figure 10. These are average values of five measurements, and the error bars represent the standard deviation. The surface of adhesive joints was the same like WC-Co carbide surface dedicated to pull-off tests and was equal to 210 mm^2^.

The results obtained from the static shear and pull-off tests confirm the effectiveness of surface modification with the use of low-temperature plasma and laser micro-texturing. In both cases, the fracture mechanism of the adhesive joints was mixed (AF+CF). The mechanical strength of the adhesive joints of WC-Co–C45 steel modified using plasma is only slightly lower than the strength that can be obtained by electrochemical etching. As described in work [34], even the use of electrolytic etching with abrasive blasting gives slightly better results—the peel strength of adhesive joints was equal to 23.9 MPa on average. However, the best result can be obtained using laser micro-texturing. The average peel strength of laser-modified WC-Co–C45 steel adhesive joints was over 24 MPa. Compared to electrochemical etching, the use of low-temperature plasma or laser processing allows a significant reduction in the processing time from 10 to 1 min, which has a large economic dimension. In addition, their advantage is also determined by ecological considerations—the lack of chemicals that may have a negative impact on the environment.

## 4. Conclusions

The proposed modern methods for modifying the surface of WC-Co carbides, grade B2, are highly effective in increasing adhesive interactions in adhesive joints. Laser processing demonstrated high efficiency, producing adhesive joints with the highest shear and peel strengths, averaging 34.5 MPa and 24.1 MPa, respectively. Moreover, it is a fully controlled process, where varying parameters can yield surfaces with the desired stereometry. In addition to the good surface development resulting from laser processing, which is beneficial for achieving strong mechanical adhesion, properties such as SFE are also improved.

The conducted research indicates that not every type of gas used for low-temperature plasma generation yields favorable results in surface modification. The best outcomes are achieved with low-temperature plasma produced from inert gasses such as Ar and He. This type of plasma has a positive effect on increasing the SFE of WC-Co carbides, making them better wetted by various liquids, including adhesives. This is important for the adhesive bonding process, as it facilitates adhesive penetration into uneven surfaces, thereby enhancing mechanical adhesion between the adhesive and the substrate. Compared to electrolytic etching, low-temperature plasma treatment does not alter surface roughness parameters and therefore does not affect surface expansion. Nevertheless, it enables the creation of adhesive joints with very similar shear and peel strength (less than a 10% difference). This is attributed to the highly clean surface and high SFE, which promote strong specific adhesion.

Achieving a shear strength above 30 MPa in adhesive joints is a noteworthy outcome, as it indicates that the joint’s strength is largely governed by the mechanical properties of the adhesive rather than the surface preparation method. It is possible to achieve greater adhesive joint strength, but this generally requires the use of composite adhesives.

Low-temperature plasma and laser treatments are more advantageous than electrochemical etching from both economic and ecological perspectives. This is due to the 10-fold reduction in processing time and the absence of chemicals that could negatively impact the environment.

## Figures and Tables

**Table 1:** Surface tension components of measuring liquids used in tests [42,44].

Measuring Liquid	Surface Tension, mN/m	Dispersion Component, mN/m	Polar Component, mN/m	Acid Parameter, mN/m	Alkaline Parameter, mN/m
Distilled water	72.8	21.8	51.0	25.5	25.5
Diiodomethane	50.8	50.8	0	0	0

**Table 2:** Surface free energy (SFE) and its components for B2 grade WC-Co carbides without and after low-temperature plasma treatment generated from various gasses.

Surface of WC-Co Carbide, Grade B2	Surface Free Energy (SFE), *γ_S_* [mJ/m^2^]	Dispersion Component, $\gamma_{S}^{d}$ [mJ/m^2^]	Polar Component, $\gamma_{S}^{p}$ [mJ/m^2^]
Without treatment	41.7	36.8	4.9
Plasma treatment—gas Ar	58.8	42.5	16.3
Plasma treatment—gas He	66.9	43.1	23.8
Plasma treatment—gas N_2_	45.4	42.2	5.1
Plasma treatment—mixture Ar + CO_2_	48.4	42.7	5.6

**Table 3:** Surface roughness parameters (Ra, Rz) of WC-Co carbides after different treatment methods.

Method of WC-Co Surface Treatment	Ra [µm]	Rz [µm]
Without treatment	1.27 ± 0.23	8.60 ± 1.21
Mechanical grinding	0.77 ± 0.41	6.28 ± 1.13
Electrolytic etching	1.68 ± 0.20	12.48 ± 0.85
Plasma treatment—gas Ar	1.25 ± 0.15	8.52 ± 0.71
Plasma treatment—gas He	1.27 ± 0.18	8.34 ± 0.87
Laser micro-texturing	3.25 ± 0.22	12.60 ± 1.85

## Data Availability

The original contributions presented in the study are included in the article, further inquiries can be directed to the corresponding author.
